# PW01-018 – Circulating endothelial biomarkers in FMF

**DOI:** 10.1186/1546-0096-11-S1-A71

**Published:** 2013-11-08

**Authors:** I Sari, BO Pamuk, S Selcuk, G Gokce, DL Kozaci

**Affiliations:** 1 Rheumatology, Dokuz Eylul University School of Medicine, Konak, Turkey; 2 Internal Medicine, Bozyaka Training and Research Hospital, Izmir, Turkey; 3 Biochemistry, Adnan Menderes University School of Medicine, Aydin, Turkey

## Introduction

Familial Mediterranean fever (FMF) is a hereditary autoinflammatory disease that affects the populations with certain ethnic backgrounds. It is characterized by self-limiting febrile attacks of polyserositis. In recent years, some studies reported that FMF patients had increased vascular wall alterations and damage which may be another clinical phenotype of the disease.

## Objectives

In the present study, we extensively evaluated biomarkers related with endothelial damage in regularly treated and attack-free FMF patients.

## Methods

Forty FMF patients and eighteen healthy controls with no known cardiovascular risk factors were included. All patients were receiving regular colchicine treatment and examinations were performed during attack-free periods. Serum samples were used for the determination of high sensitive C-reactive protein (hs-CRP), tissue factor (TF), tissue plasminogen activator (t-PA) and osteoprotegerin (OPG). Plasma samples were used for the determination of asymmetric dimethylarginine (ADMA) and thrombomodulin (TM).

## Results

There were 40 FMF patients (21 M and 19 F, 31 [15-58] years) and18 healthy subjects (11 M and 7 F, 35.5 [19-46] years). The median disease duration was 15 (0.6-45) years. Age, sex distribution, waist circumference, body mass index, smoking status and serum lipids were similar between the patients and controls (P > 0.05). The concentrations of high sensitive C-reactive protein (hs-CRP) was significantly higher in FMF patients compared to controls (hs-CRP: 0.78 [0.03-20.2] vs. 0.15 [0.02-4.71], µg/ml, P = 0.03). Asymmetric dimethylarginine (ADMA), osteoprotegerin (OPG) and thrombomodulin (TM) concentrations were significantly lower in the patients’ group compared to those of controls (ADMA: 2.56 [0.84-4.07] vs. 3.26 [0.88-3.63], umol/l, P = 0.04; OPG: 361.5 [50.5-1232] vs. 548.9 [193-1181], pg/ml, P = 0.01; TM: 2.69 [0.92-7.26] vs. 3.59 [2.8-8.3], ng/ml, P = 0.001 respectively). However, von Willebrand factor (vWF), tissue factor (TF) and tissue plasminogen activator (t-PA) levels were similar between the groups (P > 0.05).

## Conclusion

In this study we showed that markers related with endothelial injury including ADMA, OPG and TM were significantly down-regulated in FMF patients who were on regular colchicine treatment during attack-free disease state.

## Disclosure of interest

None declared

